# Mapping and functional verification of leaf yellowing genes in watermelon during whole growth period

**DOI:** 10.3389/fpls.2022.1049114

**Published:** 2022-10-19

**Authors:** Yingchun Zhu, Gaopeng Yuan, Yifan Wang, Guolin An, Weihua Li, Junpu Liu, Dexi Sun

**Affiliations:** ^1^ The Key Laboratory of Genetic Resource Evaluation and Application of Horticultural Crops (Fruit), Ministry of Agriculture, Zhengzhou Fruit Research Institute, Chinese Academy of Agricultural Sciences, Zhengzhou, China; ^2^ Western Research Institute, Chinese Academy of Agricultural Sciences, Changji, China

**Keywords:** Watermelon, leaf yellowing, gene mapping, fragment deletion, gene function

## Abstract

Increasing light energy utilization efficiency is an effective way to increase yield and improve quality of watermelon. Leaf is the main place for photosynthesis, and the color of leaf is directly related to the change of photosynthesis. In addition, leaf yellowing can be used as a marker trait to play an important role in watermelon hybrid breeding and improve seed breeding. It can not only be used to eliminate hybrids at seedling stage, but also be used to determine seed purity. In this study, transcriptome analysis was first carried out using the whole growth period leaf yellowing watermelon mutant *w-yl* and inbred line ZK, and identified 2,471 differentially expressed genes (DEGs) in the comparison group *w-yl*-vs-ZK. Among the top 20 terms of the gene ontology (GO) enrichment pathway, 17 terms were related to photosynthesis. KEGG pathway enrichment analysis showed that the most abundant pathway was photosynthesis—antenna proteins. The F_2_ population was constructed by conventional hybridization with the inbred line ZK. Genetic analysis showed that leaf yellowing of the mutant was controlled by a single recessive gene. The leaf yellowing gene of watermelon located between Ind14,179,011 and InD16,396,362 on chromosome 2 by using indel-specific PCR markers, with a region of 2.217 Mb. In the interval, it was found that five genes may have gene fragment deletion in *w-yl*, among which *Cla97C02G036010*, *Cla97C02G036030*, *Cla97C02G036040*, *Cla97C02G036050* were the whole fragment loss, and *Cla97C02G0360* was the C-terminal partial base loss. Gene function verification results showed that *Cla97C02G036040*, *Cla97C02G036050* and *Cla97C02G036060* may be the key factors leading to yellowing of *w-yl* leaves.

## Introduction

Photosynthesis is essential in the process of plant growth and development, which is of great significance for plant survival. Leaves are the main place for photosynthesis in plants, and leaf color determines photosynthetic efficiency to a large extent (Chen et al., 2022). Different pigments can absorb light waves of different lengths, so leaves of plants show different colors due to different pigment contents and proportions. Leaf color mutation is a frequent and easily recognized phenomenon in nature, so leaf color mutants are ideal materials for studying plant development (Yuan et al., 2022b). At present, mutant materials have been found in a variety of plants, and the leaf color mutation types include albino, etiolation, stripe, yellow-green, green-yellow, green-white, light green and verdant green, etc (Awan et al., 1980). There are many ways of forming leaf color mutation. External factors mainly include light, temperature, plant hormones, mineral elements and metal ions. Internal factors mainly include genes related to photosynthetic pigment metabolism pathway, such as chloroplast biosynthesis pathway, chlorophyll degradation pathway, heme metabolism pathway and carotenoid metabolism pathway; as well as genes related to chloroplast development, such as chloroplast development and protein synthesis, nucleoplasmic interactions. All of these can lead to a decrease in the chlorophyll content of plant leaves, resulting in the leaves can not appear green color (Zhang et al., 2006; Sugliani et al., 2016; Li et al., 2018).

Studies on leaf color mutations mainly focus on the cell structure, photosynthetic physiology, molecular biology and other aspects of leaf color mutants, among which more in-depth studies have been conducted in model plants such as rice and *Arabidopsis*. For example, more than 160 leaf color mutants have been found in rice, distributed on 12 chromosomes, among which a small number of leaf color mutants have been cloned (Dong et al., 2013; Huang et al., 2017; Tan et al., 2019). Among them, 14 genes are directly involved in chlorophyll biosynthesis and catabolism (Sakuraba et al., 2013), and 6 genes are indirectly involved in this process (Yang et al., 2011), while 16 genes are directly involved in chloroplast development regulation (Gothandam et al., 2005) and 3 were indirectly involved in this process (Hibara et al., 2009) Therefore, the mutant genes are mainly divided into two categories, namely, genes in the chlorophyll biosynthesis and degradation pathway and genes in the chloroplast development pathway. In addition, previous studies have proved that most leaf color mutations are nuclear inheritance except for a small number of leaf color mutations for cytoplasmic inheritance (Kong et al., 2016; Li et al., 2021a; Li et al., 2021b). In recent years, with the application of high-throughput sequencing, the study of leaf color mutation has been gradually carried out in some important economic crops and ornamental plants, such as tea, pepper, maize, melon and cucumber (Shao et al., 2013; Li, 2016; Lai et al., 2018; Wang et al., 2019; Zhu et al., 2019; Gao et al., 2020; Xiong et al., 2020), which will help improve crop quality and increase yield (Shao, 2013; Ren et al., 2019). The results of the latest study on cucumber showed that the post-green mutant *SC311Y* was controlled by a recessive gene, which was identified as the gene controlling chloroplast development by BSA-seq and RNA-seq techniques (Zhang et al., 2022).

The genetic basis of watermelon is narrow and the natural mutation rate is low. There are few studies on watermelon leaf color mutants. The leaf color mutation materials are mainly divided into four categories: (1) watermelon leaf color mottled mutants, which are characterized by white-green cotyledons and mosaic-like spots in the first true leaf under low temperature environment (Provvidenti, 1994; Wang et al., 2011); (2) watermelon albino mutant, showing pale yellow or pale cream cotyledons, gradually turning green but remaining white at leaf margins, white tendrils, petioles, petals and hypocotyls (Zhang et al., 1996; Wang et al., 2011; Hou et al., 2016); (3) incomplete dominant yellow leaf mutants (Hou et al., 2016); (4) In post-green mutants, the leaves showed light green cotyledons and leaves at the early stage, and changed to normal green at the later stage (Wang and Wang, 1997; Ma and Zhang, 1999; Wang et al., 2011; Xu et al., 2022). In terms of genetic analysis and molecular biology, the early stage mainly focused on the study of genetic patterns, and confirmed that watermelon leaf color mutants were controlled by recessive genes based on the discovered mutant materials (Rhodes, 1986; Provvidenti, 1994; Zhang et al., 1996). With the publication of watermelon genome and the rapid development of sequencing technology (Guo et al., 2019; Wu et al., 2019), more high density genetic maps of watermelon emerged (Duan et al., 2022), but only a few maps involved watermelon leaf color. For example, Haileslassie (Haileslassie, 2020) found the presence of a SNP in the gene *ClCG03G010030* of the watermelon post-green mutant *Houlv*, resulting in an arginine to lysine mutation. The gene encodes an FtsH extracellular protease family protein which is involved in the development of early chloroplast. Exploring the mechanism of leaf color variation can provide a theoretical basis for genetic improvement and meet people’s needs in production, seed selection and breeding.

China is the largest watermelon planting and consumption country in the world. Although the demands for watermelon is diversified, cultivating new varieties with high yield and high quality is still the main direction of watermelon breeding. Improving the utilization efficiency of light energy of watermelons is an effective way to promote yield and improve quality. In this study, yellow leaf throughout the whole growth period material *w-yl* and green leaf material ZK were used as experimental materials. The position of the leaf yellowing gene in the chromosome was preliminarily located by BSA-seq technology. The high-density genetic map was constructed by the F_2_ population using InDel markers for mapping the position of the mutant gene in the chromosome, and the key candidate genes and key variations were screened in combination with transcriptome data. Finally, the virus-induced gene silencing (VIGS) assay was performed on the key candidate genes to clarify the function of the yellowing leaf gene. The development of this study will help to explore the mechanism of leaf yellowing in the whole growth period of watermelon, and provide theoretical support for the application of leaf yellowing and molecular marker-assisted selection of new watermelon varieties with high photosynthetic efficiency.

## Materials and methods

### Plant material cultivation and samples collection

The leaf color yellowing mutant material *w-yl* was obtained from the National Mid-term Genebank for Watermelon and Melon (Zhengzhou, China), the leaves in the whole growth period were yellow, including cotyledon and fruit. Normal green leaf material ZK was supplied by the Diploid Watermelon Genetics and Breeding Research Group of Zhengzhou Fruit Research Institute (ZZFRI) of Chinese Academy of Agricultural Sciences (CAAS). In this study, the mutant material was crossed with the ZK, and six generations were constructed: P_1_ (the yellow parent *w-yl*), P_2_ (the green leaf parent ZK), F_1_ (orthogonal), BC_1_P_1_, BC_1_P_2_, and F_2_. The materials were planted in a greenhouse at the Xinxiang Comprehensive Experimental Base of CAAS, with a row spacing of 1.5 m and a plant spacing of 0.4 m. The phenotype of leaf color was determined by visual observation.

Plant for chlorophyll were planted in an artificial climate chamber and treated with different environmental factors at three true-leaf stage: temperature 35°C/28°C, light intensity 30,000 Lx, namely HTHL(high temperature and high light); temperature 35°C/28°C, light intensity 12,000 Lx, namely as HTNL (high temperature and normal light); temperature 35°C/28°C, light intensity 5,000 Lx, marked as HTLL (high temperature and low light); temperature 28°C/25°C, light intensity 30,000 Lx, marked as NTHL (normal temperature and high light); temperature 28°C/25°C, light intensity 12,000 Lx, marked as NTNL (normal temperature and normal light); temperature 28°C/25°C, light intensity 5,000 Lx, marked as NTLL (normal temperature and low light); temperature 15°C/15°C, light intensity 30,000 Lx, marked as LTHL (low temperature and high light); temperature 15°C/15°C, light intensity 12,000 Lx, marked as LTNL (low temperature and normal light); 15°C/15°C, 5,000 Lx, labeled as LTLL (low temperature and low light). Light cycle was 16h/8h, humidity 80%. Each treatment set three replicates. Chlorophyll content was determined after 8 days of treatment.

Plant for chlorophyll precursors and transcriptome sequencing were grown in a smart greenhouse in ZZFRI of CAAS, the light cycle was 16h/8h, the temperature was 25°C/18°C and the light is natural light. The leaves were sampled after 8 days of treatment.

### Determination of pigment content

The third true leaf from five seedlings was sampled and mixed, weighed 0.1 g and put into a 15 mL centrifuge tube respectively, added 10 mL of 96% ethanol, and soaked in dark environment until the leaves turned completely white (Yuan et al., 2017). The absorbance A665, A649 and A470 at 665 nm, 649 nm and 470 nm were determined by UV spectrophotometer (UV-2600I, Shimadzu, Kyoto, Japan). The concentrations of chlorophyll a (chla), chlorophyll b (chlb), total chlorophyll (chla+b) and carotenoids were calculated using 96% ethanol as blank control. The equations are as following:


$$Chla\;\left(mg\cdot L^{-1}\right)\;=\;13.95\;\times \;A_{665}-6.88\;\times \;A_{649}$$

$$Chlb\;\left(mg\cdot L^{-1}\right)\;=\;24.96\;\times \;A_{649}-7.32\;\times \;A_{665}$$

$$Chla+b\;\left(mg\cdot L^{-1}\right)\;=\;chla+chlb\;=\;6.63\;\times \;A_{665}+18.08\;\times \;A_{649}$$

$$Carotenoids\;\left(mg\cdot L^{-1}\right)\;=\;\left(1000\;\times \;A_{470-}2.05\;\times \;Chla\;-114.8\;\times \;Chlb\right)/248$$

Chlorophyll content (mg·g^-1^ = (C × V)/(W × 1000). C represents chlorophyll content, V represents the total volume of extract (mL), and W represents leaf mass (g).

### Determination of chlorophyll precursor

The contents of main chlorophyll precursor in the process of chlorophyll synthesis were measured, among which δ-aminolevulinic acid (ALA) was determined according to the method of Dei (Dei, 2010) and the molar concentration of ALA was calculated with a molar extinction coefficient of 7.2 × 10^4^ mol^-1^·cm^-1^at 535 nm. Relative contents of protoporphyrin IX (protoIX), Mg-protoporphyrin IX (Mg-proto IX), and pchlide were determined according to the method of Rebeiz (Rebeizjames et al., 1975) and Lee (Lee et al., 1992). The relative mass molar concentration of Mg-Proto IX is presented as F $\frac{440}{ex595}$ : fluorescence emission intensity at 595 nm under 440 nm excitation light. Proto IX $\left(F\frac{440}{ex633}\right)$ ) = $(F\;\frac{440}{ex633}\;-0.25\;\times \;F\;\frac{440}{ex622}\;-0.24\;\times \;F\;\frac{440}{ex640})/0.95;$ $Pchlide\text{ (}\frac{440}{ex640})=\;(F\;\frac{440}{ex640}\;-0.03\;\times \;F\;\frac{440}{ex633})/0.99.$

### RNA sequencing

Leaves of five plants were selected as a sample from *w-yl* and ZK, with three biological replicates respectively. Total RNA was extracted using RNeasy Plant Mini Kit (Beijing Tiagen), following the manufacturer’s instructions. Then RNA was reversely transcribed to cDNA, and the cDNA fragments were segmented by PCR. Finally, the double-stranded PCR product is thermally denatured to form single-stranded circular DNA, which is then formatted into a final library. The cDNA library was sequenced by BGISEQ-500 system (BGI-Shenzhen, China) with reads of 100bp in length.

The sequencing data were screened to obtain Clean reads, which were then mapped into the ‘97103’ watermelon genome (http://cucurbitgenomics.org/organism/21) using Bowtie2. Gene expression levels were calculated using FPKM (million fragments per kilobase). Based on KEGG (http://www.genome.jp/kegg/) and GO (http://www.geneontology.org/) database for gene annotation and function assignment. Differentially expressed genes (DEGs) were set as gene fold change ≥2.00 and false discovery rate ≤0.001. Through GO enrichment and KEGG enrichment pathways, the significantly enriched metabolic pathways were screened and compared with the whole genome background. Functional classification of DEGs was performed according to GO and KEGG annotation results and official classification, and FDR ≤ 0.01 was set as significant enrichment.

### QPCR validation and gene expression analysis

Total RNA was extracted by plant RNA kit (Huayue Yang Biotechnology Co., LTD.). A total of 1.0 μg of RNA was used for cDNA synthesis using the PrimeScript RT kit and gDNA Eraser (TaKaRa) according to the manufacturer’s protocol. Primers were designed using NCBI online tools (https://www.ncbi.nlm.nih.gov/tools/primer-blast/), and synthesized by Sangon Biotech (Shanghai, China). All the primer sequences were shown in **Supplementary Table 1**. Quantitative real-time PCR reaction procedure and system were as described previously (Yuan et al., 2022a). All primers are shown in **Supplementary Table S1**. The 2^−ΔΔCt^ method was used to calculate relative gene expression values (Kenneth and Thomas, 2002).

### BSA-seq analysis of the leaf yellowing genes

Leaf DNA of 30 individual plants with yellowed and green extreme phenotypes in F2 population were selected for the construction of two extreme sequencing mixed pools, and parental DNA was used to construct the parental pools for sequencing analysis. The depth of parental sequencing was 20×, and the depth of extremely mixed-pool sequencing was 30×. Sequencing was performed by Biomarker Technologies Co, LTD (Beijing, China) using Illumina HiSeq2000. The sequencing read length was 150 bp.

Raw reads were filtered to remove reads containing adapter, and reads containing >5% N and low-quality reads (the number of bases with quality value Q ≤ 10 accounted for more than 50% of the whole read) were used to obtain clean reads for subsequent analysis. Clean reads were mapped to the ‘97103’ watermelon genome (http://cucurbitgenomics.org/organism/21) using BWA software. Then GATK (4.0.4.0) and SNPeff (4.3) were used to annotate the mutation sites, and single nucleotide polymorphisms (SNPs) and insertion-deletion polymorphisms (InDels) were identified.

The SNP-index algorithm was used to establish the target region to find the significant difference in genotype frequency between the pool, and Δ(SNP-index) was used for statistics. In this project, the DISTANCE method was used to fit the ΔSNP-index, and then the region above the threshold was selected as the region related to the trait according to the association threshold. The stronger association between SNP and trait, the closer Δ(SNP-index) to 1.

### Functional analysis of key genes

Using the cDNA of green leaf ZK as template, specific primers were designed to amplify the CDS regions of *Cla97C02G036010*, *Cla97C02G036030*, *Cla97C02G036040*, *Cla97C02G036050* and *Cla97C02G036060*, and the primers were shown in **Supplementary Table 1**. BamHI (GGATCC) restriction sites were added to both ends of the primers and inserted into the cucumber green mottle mosaic virus (CGMMV) gene silencing vector PV190 by homologous recombination to construct virus-induced gene silencing (VIGS) vector. The dual vector was transformed into *Agrobacterium tumefaciens* GV3101.

Induction and inoculation of *A. tumefaciens* according to Liu (Liu, 2019) When watermelon seedlings were at cotyledon stage, the induced *A. tumefaciens* was injected from the back of watermelon cotyledon with 1 mL syringe. The blank control (Blank, B), water control (Water, W), medium control (YT medium, Y), blank vector control (PV190, P) and *PDS* gene positive control (PDS) were set up respectively. Three biological replicates were set up for each treatment. Two weeks after injection, leaf phenotype was observed, and samples were collected for ultrastructural analysis, chlorophyll content measurement and gene expression analysis.

### Ultrastructural observation of chloroplast

The above-mentioned leaves with phenotype after *A. tumefaciens* were used as materials, fixed with 4% glutaraldehyde (configured with pH 7.2 phosphate buffer) overnight at 4°C, rinsed with phosphate buffer three times, fixed with 1% osmium tetroxide for 1 h, rinsed with phosphate buffer three times, dehydrated with 30%, 50%, 70%, 80%, 95%, 100% ethanol and acetone step by step for 5 min, and finally embedded with resin. After sectioning, they were stained with 2% uranyl acetate saturated alcohol and lead citrate for 15 min, and the chloroplast ultrastructure was observed under transmission electron microscope (HT7700, Hitachi, Japan).

### Data statistical analysis

All data graphs were analyzed by Office 2016 software. Differences were analyzed by SPSS 18.0 software, and one-way ANOVA was used for statistical analysis, p< 0.05 (n = 3) was considered significant difference.

## Results

### Genetic characteristics analysis of yellowing leaf color

**Figure 1:**
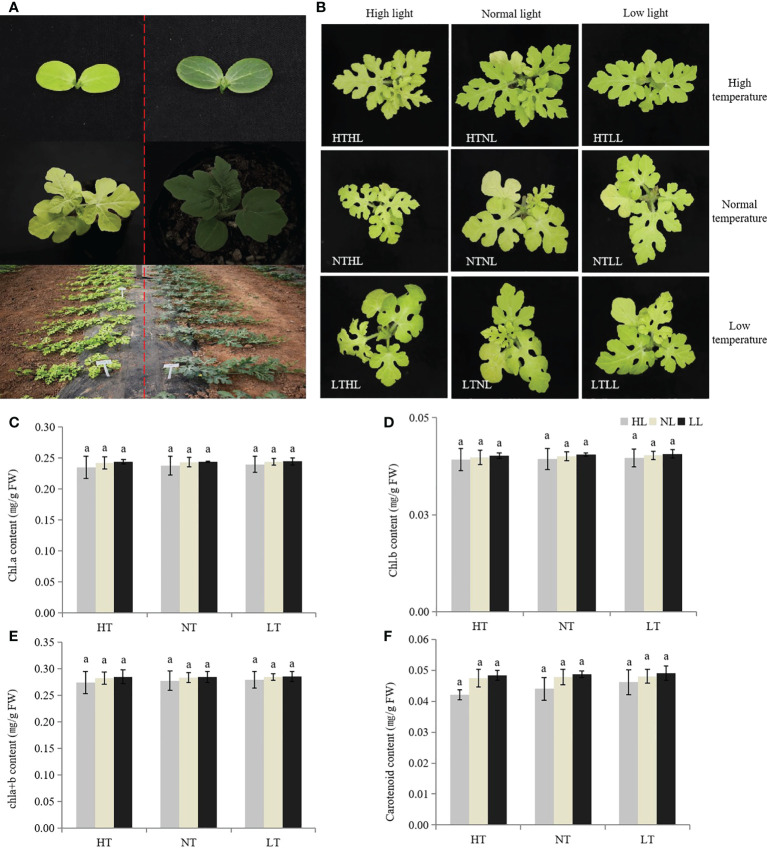
Plant phenotypes of w-yl and ZK. **(A)** Plant phenotypes of w-yl (left) and ZK (right) at different developmental stages. **(B)** Plant phenotypes of w-yl under different temperature and light intensity. The content of **(C)** chla, **(D)** chl b, **(E)** chla+b and **(F)** carotenoid under different temperature and light intensity. Small letters represent significant difference at P<0.05.

The leaves of *w-yl* showed yellow throughout the whole growth period (**Figure 1A**), and the color did not change with environmental changes, such as temperature and light intensity (**Figure 1B**). Under different temperature and light intensity, there were no significant differences in the contents of chla, chlb, chla+b and carotenoids.

In addition, phenotypic data showed that all F_1_ plants appeared green leaves, indicating that the yellow mutation was recessive. For F_2_ plants, among the 237 progeny in the summer of 2018, 178 plants had green leaves and 59 plants had yellow leaves; among the 993 progeny in spring of 2019, 730 had green leaves and 263 had yellow leaves (**Table 1**). The χ^2^ test of green and yellow leaves in the two seasons showed that the separation pattern was consistent with the Mendelian separation ratio of 3:1 (χ^2^ > χ^2^_0.05_ = 3.841). Furthermore, for the backcross progeny BC_1_P_1_ and BC_1_P_2_, the yellow leaf plants were 29 and 0 respectively, indicating that the yellowing mutation of watermelon leaves conformed to the genetic pattern controlled by a single recessive nuclear gene, and green leaves were dominant to yellowing.

**Table 1:** Phenotype of yellow mutant to green leaf trait and Chi-square goodness-fit test ratios in different populations.

Population	Number	Green leaves	Yellow leaves	Expected ratio		P value
P_1_	15		15			
P_2_	15	15				
F_1_	30	30				
F_2_ (Summer of 2018)	237	178	59	3:1	0.0014	0.9701
F_2_ (Spring of 2019)	993	730	263	3:1	1.1685	0.2797
BC_1_P_1_	54	29	25	1:1	0.2963	0.5862
BC_1_P_2_	30	30	0			

### Genetic characteristics analysis of yellowing leaf color

**Figure 2:**
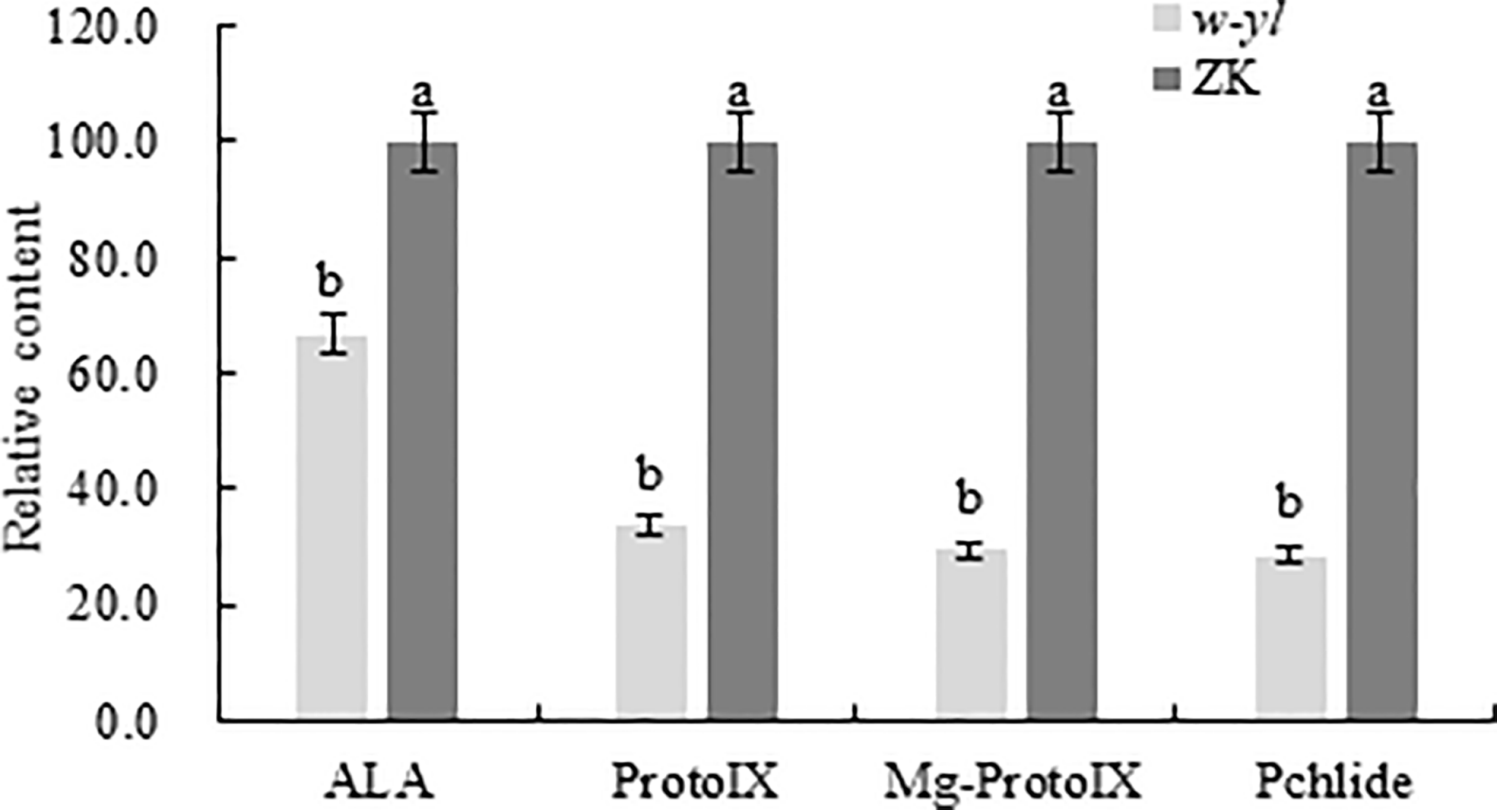
Chlorophyll precursors content of the *w-yl* and CK in pepper leaf. Small letters represent significant difference at P<0.05.

Previous studies had demonstrated that there are significant differences in chlorophyll content and photosynthetic indicators (Ren et al., 2019). To further validate the difference, the chlorophyll precursors, including ALA, protoIX, Mg-ProtoIX and pchlide were analyzed (**Figure 2**). The results showed that the contents of four indexes detected in the *w-yl* were significantly lower than those in ZK, which explained the low chlorophyll content to a certain extent.

### RNA-seq for the leaves of *w-yl* and ZK

**Figure 3:**
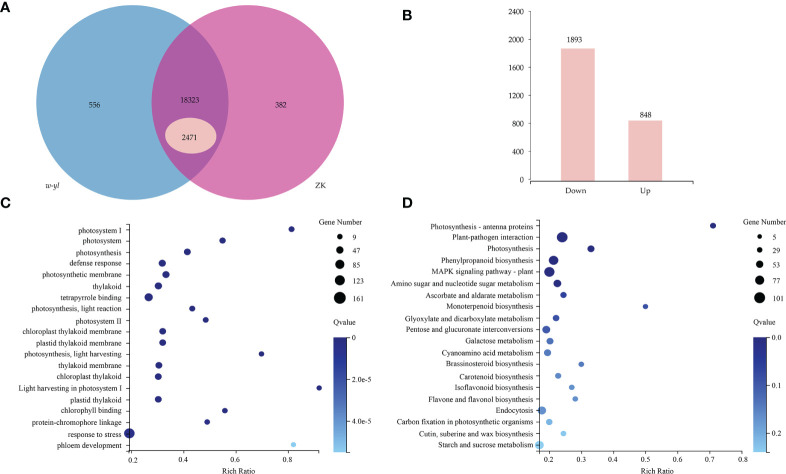
RNA-seq analysis for the leaves of *w-yl* and ZK. **(A)** Venn diagram of the relationship of *w-yl-*vs-ZK. **(B)** Number of up-regulated and down-regulated DEGs. **(C)** GO enrichment analysis of DEGs. **(D)** KEGG pathenrichment analysis of DEGs.

A total of 6 samples were measured by RNA-seq, including 3 samples for *w-yl* and 3 samples for ZK, yielding an average of 6.06 Gb of data per sample. The average rate of genome alignment was 89.40%, and the average rate of gene set alignment was 65.81% (**Figure 3A**). For the comparison group *w-yl*-vs-ZK, a total of 19,261 genes were detected, and there were 18,323 shared genes, including 2,471 DEGs (**Figure 3A**), with 848 up-regulated DEGs and 1893 down-regulated DEGs (**Figure 3B**).

GO enrichment and KEGG pathway enrichment analyses were carried out to better understand the function of DEGs. For GO enrichment, 17 of the top 20 selected GO terms were related to photosynthesis process, including 4 terms related to photosystem, such as photosystem (GO:0009521), photosystem I (GO:0009522), Photosystem II (GO:0009523), light harvesting in photosystem I (GO:0009768); 4 terms involved in photosynthesis, such as photosynthesis (GO:0015979), photosynthetic membrane (GO:0034357), photosynthesis—light reaction (GO:0019684) and photosynthesis—light harvesting (GO:0009765); 6 terms involved in thylakoid, such as thylakoid (GO:0009579), thylakoid membrane (GO:0042651), chloroplast thylakoid (GO:0009534), chloroplast thylakoid membrane (GO:0009535), plastid thylakoid (GO:0031976) and plastid thylakoid membrane (GO:0055035); 3 terms relate to pigments, such as tetrapyrrole binding (GO:0046906), chlorophyll binding (GO:0016168) and protein-chromophore linkage (GO:0018298) (**Figure 3C**; **Supplementary Table S2**). These results showed that *w-yl* and ZK had significant differences in photosynthesis.

**Figure 4:**
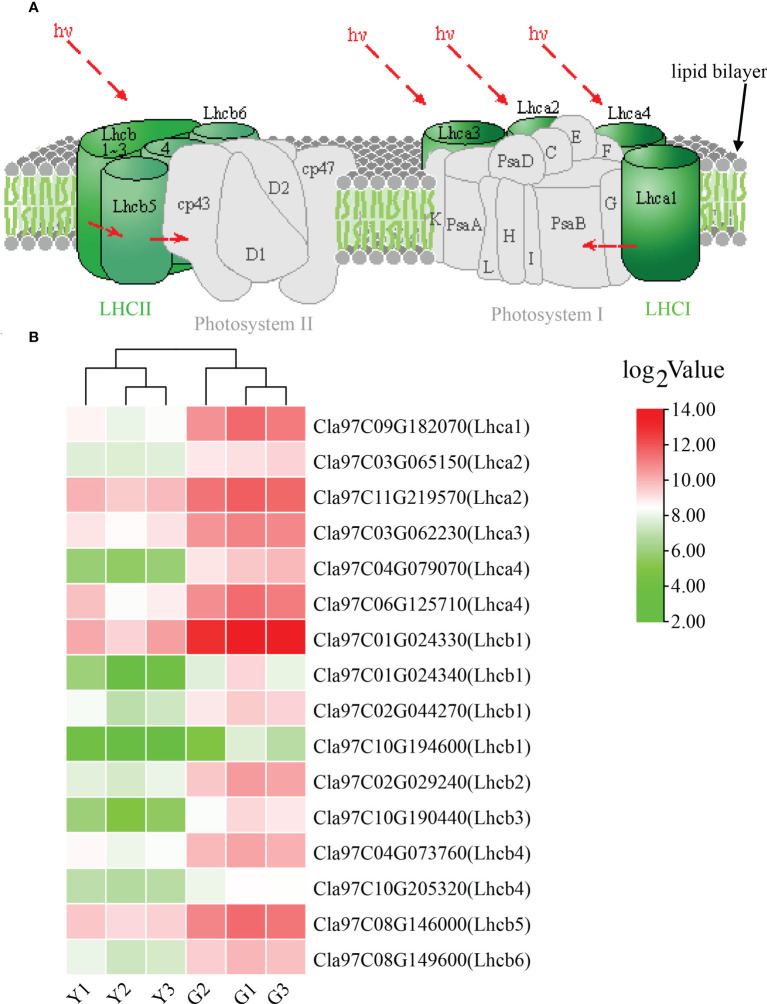
Analysis of the photosynthesis—antenna proteins. **(A)** Role of photosynthesis—antenna proteins in photosynthetic system. **(B)** Expression of photosynthesis—antenna proteins related DEGs.

For KEGG pathway enrichment, the two most significant enrichment pathways of the top 20 pathways were photosynthesis—antenna proteins and plant—pathogen interaction (**Figure 3D**; **Supplementary Table S3**). Due to the importance of antenna protein for photosynthesis (**Figure 4A**), we focused on the analysis of antenna protein-related DEGs, and completely screened 16 DEGs that encoded antenna protein (**Figure 4B**). LHCI and LHCII, as important components of photosystem I complex and photosystem II complex, are composed of four and six small components, respectively. For LHCI, the number of DEGs that encoded LHCI Chl a/b binding protein 1 (Lhca1), Lhca2, Lhca3 and Lhca4 was 1, 2, 1 and 2, respectively. For LHCII, the number of DEGs that encoded LHCII Chl a/b binding protein 1 (Lhcb1), Lhcb2, Lhcb3, Lhcb4, Lhcb5 and Lhcb6 was 4, 1, 1, 2, 1 and 1, respectively. The expression levels of all the 16 DEGs in ZK were significantly higher than those of *w-yl*, and the fold change was between 2.6 and 14.0 (**Figure 4B**).

### Verification of DEGs of qPCR and RNA-seq data

**Figure 5:**
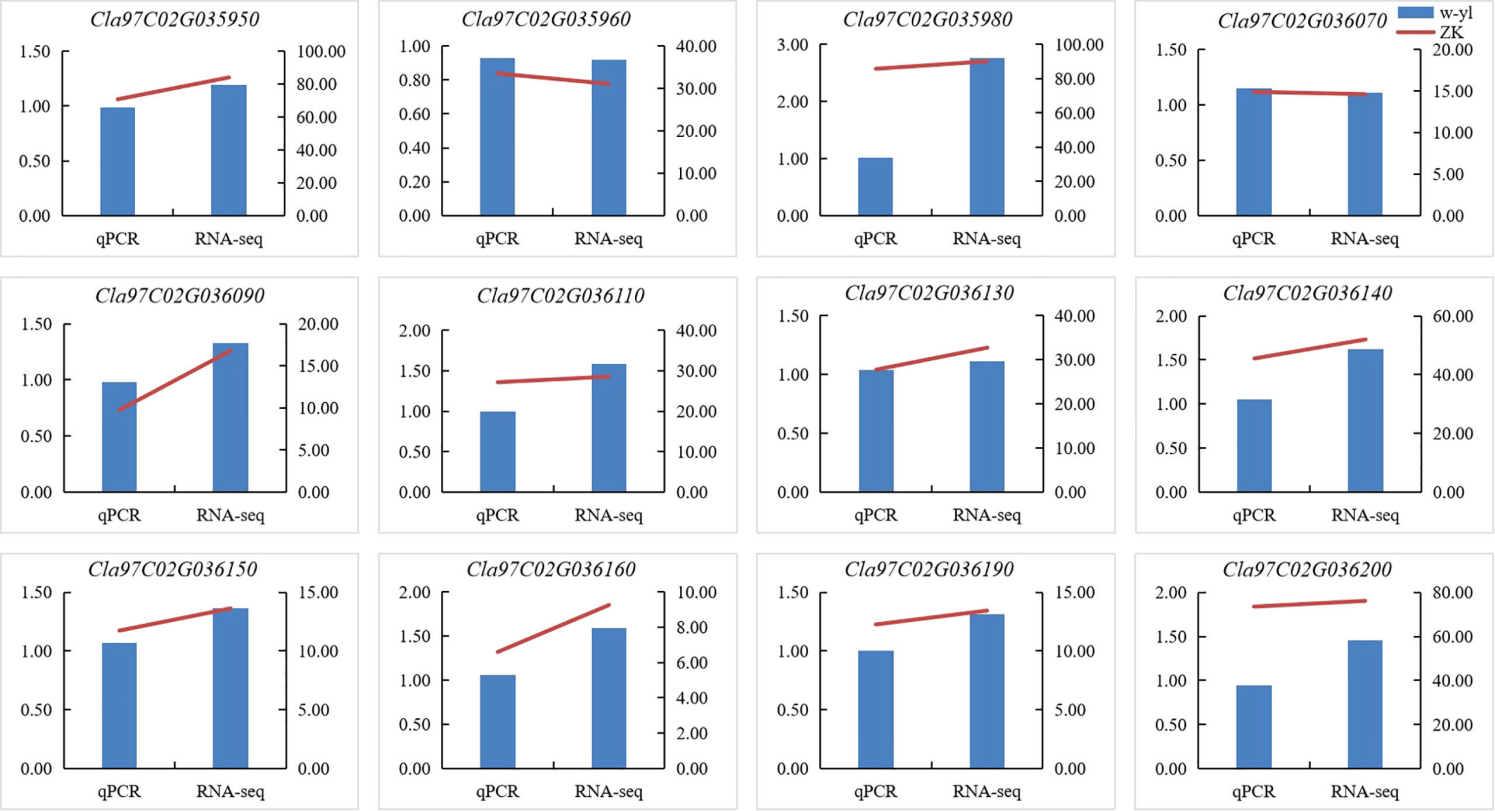
Verification of DEGs by RT-qPCR.

To verify the accuracy of RNA-seq data, 12 DEGs (*Cla97C02G035950*, *Cla97C02G035960*, *Cla97C02G035980*, *Cla97C02G036070*, *Cla97C02G036090*, *Cla97C02G036110*, *Cla97C02G036130*, *Cla97C02G036140*, *Cla97C02G036150*, *Cla97C02G036160*, *Cla97C02G036190* and *Cla97C02G036200*) of the 29 genes in the interval were selected to conduct qPCR (**Figure 5**). The results showed that expression patterns of 12 DEGs were highly consistent with those of genes in RNA-seq data, which demonstrated that the RNA-seq data are reliable.

### Mapping of yellowing gene in w-yl leaf

**Figure 6:**
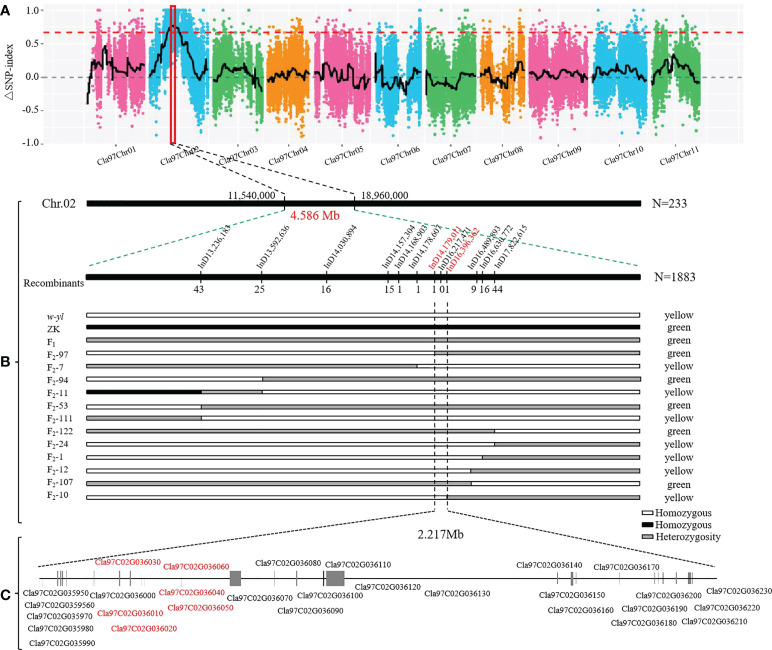
Location of yellowing gene on watermelon chromosome 2. **(A)** Δ(SNP-index) of watermelon chromosomes. **(B)** The candidate genes was mapped to a 4.586 Mb region between InD14,179,011 and InD16,396,362 on chromosome 2. **(C)** Putative genes in the candidate region based on the watermelon reference genome annotation.

In order to quickly identify the key candidate genes related to leaf color in the F_2_ population, 30 green and 30 yellow leaf progeny were selected and sequenced on the Illumina platform. A total of 51.0 Gb clean bases were generated with an average depth of about 26.5×. Finally, we identified 266,255 SNPs between *w-yl* and ZK, and 83,373 SNPs between the F_2_ pools. According to the SNP-index values of *w-yl* and ZK, the Δ(SNP-index) value of approximately 7.42 Mb genome region (11,540,000-18,960,000) on chromosome 2 was greater than the threshold (**Figure 6A**). These results indicated that this region might contain the key gene of watermelon leaf yellow traits.

In order to further locate the candidate genes for yellowing leaf, the chromosome region of the variation between *w-yl* and ZK were analyzed. A total of 12 pairs of InDel molecular markers (**Supplementary Table S4**) were developed for the candidate region based on 233 F_2_ populations. The results verified that these genes were in the range of 11.54 Mb—18.96 Mb on chromosome 2. Subsequently, based on the determination of leaf color phenotype data and individual exchange genotype, 12 recombinant individuals were further screened using 1883 F_2_ populations. Finally, it is found that the candidate interval corresponds to the 2.217 Mb region of InD14,179,011—InD16,396,362 (**Figure 6B**). There were 29 genes in this region and annotated them according to the watermelon reference genome (**Figure 3C**; **Table 2**). Notably, compared with ZK, *Cla97C02G036010*, *Cla97C02G036020*, *Cla97C02G036030*, *Cla97C02G036040*, and *Cla97C02G036050* were completely absent in *w-yl*, and *Cla97C02G036060* had partial base deletion, suggesting that they were the key genes determining *w-yl* leaf color mutation (**Supplementary Figure S1**).

**Table 2:** Gene function annotation information in candidate interval.

Gene name	Gene function
*Cla97C02G035950*	Translator-related TMA7
*Cla97C02G035960*	BZIP transcription factor, putative (DUF1664)
*Cla97C02G035970*	lipid-binding serum glycoprotein
*Cla97C02G035980*	Protein nucleo-fusion transmitter 6, chloroplast/mitochondria-like isoform X1
*Cla97C02G035990*	Unknown protein
*Cla97C02G036000*	L-ascorbate oxidase homolog, Oxidoreductase activity, Cu^2+^ binding
*Cla97C02G036010*	Unknown protein
*Cla97C02G036020*	Two component response regulator like protein
*Cla97C02G036030*	Transmembrane protein, putative
*Cla97C02G036040*	Protein containing DUF679 domain
*Cla97C02G036050*	DnaJ homologous subfamily B member 13 like
*Cla97C02G036060*	Protein Ycf2
*Cla97C02G036070*	U11/U12 small ribonucleoprotein 65 kDa protein isoform X2
*Cla97C02G036080*	Unknown protein
*Cla97C02G036090*	RING-type E3 ubiquitin transferase
*Cla97C02G036100*	family proteins containing pentapeptide repeats
*Cla97C02G036110*	Niemann-Pick C1 protein-like isoform X2
*Cla97C02G036120*	Zinc finger family protein
*Cla97C02G036130*	Integral hemolysin III-like protein
*Cla97C02G036140*	Ser/Thr-rich T10 in the DGCR region
*Cla97C02G036150*	Phosphoglycerate mutagenase family proteins
*Cla97C02G036160*	SEC1 family transporter SLY1, Oxidoreductase activity, Mg^2+^ binding
*Cla97C02G036170*	Unknown protein
*Cla97C02G036180*	Retrotransposon protein, unclassified
*Cla97C02G036190*	Glycine-rich RNA-binding protein, putative
*Cla97C02G036200*	Plant UBX domain protein 4
*Cla97C02G036210*	Calcium-permeable stress-gated cation channel 1
*Cla97C02G036220*	Acid phosphatase/vanadium-dependent haloperoxidase-related protein
*Cla97C02G036230*	Core-2/I branch β-1,6-N-acetylglucosamine aminotransferase family proteins

**Figure 7:**
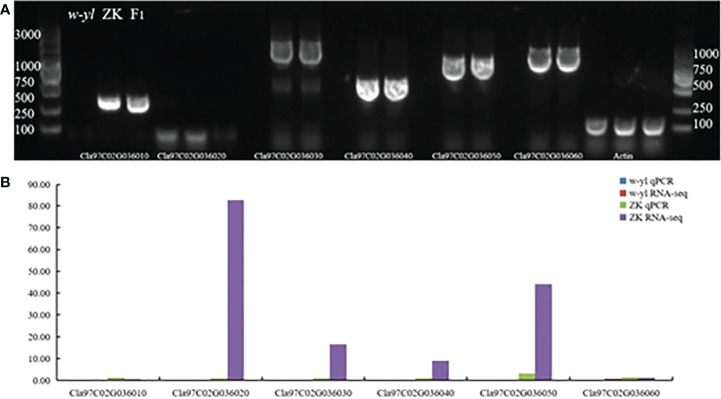
Amplification of candidate genes *Cla97C02G036010, Cla97C02G036020, Cla97C02G036030, Cla97C02G036040, Cla97C02G036050* and *Cla97C02G036060*. **(A)** Agarose gel electrophoresis analysis of candidate genes. **(B)** RNA-seq and qPCR analysis of candidate genes.

In addition, the results of agarose gel electrophoresis and qPCR showed that *Cla97C02G036010, Cla97C02G036020, Cla97C02G036030, Cla97C02G036040, Cla97C02G036050* and *Cla97C02G036060* could not be amplified in *w-yl*, as well as *Cla97C02G036020* also had no target product in ZK (**Figure 7**). The RNA-seq results also showed the same results (**Figure 6C**). These results further proved the importance of *Cla97C02G036010, Cla97C02G036030, Cla97C02G036040, Cla97C02G036050* and *Cla97C02G036060* in leaf yellowing.

### Function analysis of yellowing gene in ZK leaf

**Figure 8:**
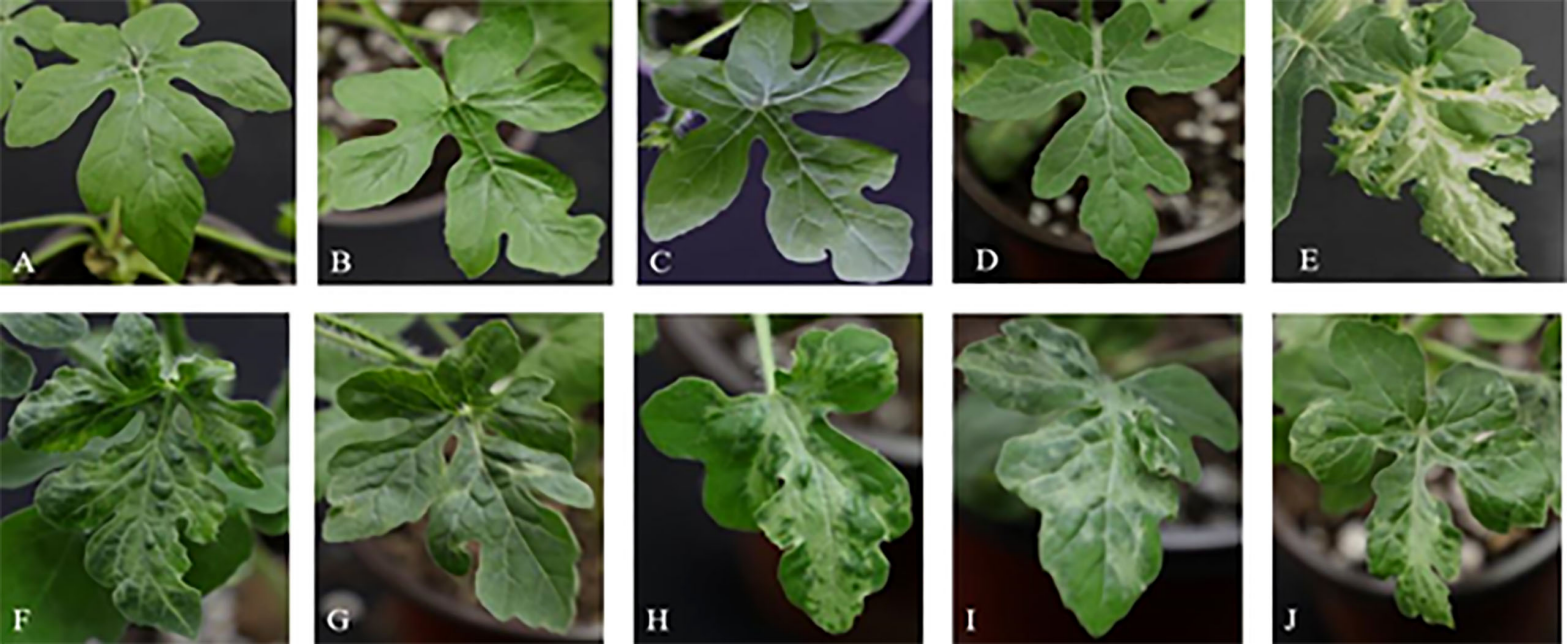
Leaf phenotypic analysis of candidate gene silencing. Phenotype of **(A)** blank control, **(B)** water control, **(C)** medium control, **(D)** blank vector control, **(E)** positive control, **(F)** silencing *Cla97C02G036010*, **(G)** silencing *Cla97C02G036030*, **(H)** silencing *Cla97C02G036040*, **(I)** silencing *Cla97C02G036050*, **(J)** silencing *Cla97C02G036060*.

In order to verify the gene function of the candidate genes, cucumber mosaic virus-mediated VIGS vector was used to perform gene silencing assay on ZK leaves. The results showed that at 16 days after inoculation (DAI), the plants inoculated with water (**Figure 8B**), medium (**Figure 8C**) and blank vector (**Figure 8D**) showed no significant difference in phenotype compared with the blank control (**Figure 8A**), while the positive control plants inoculated with *PDS* gene showed virus symptoms at DAI16, with severe true leaf pucking and chlorosis (**Figure 8E**). Watermelon plants silencing *Cla97C02G036010* (**Figure 8F**) and *Cla97C02G036030* (**Figure 8G**) showed symptoms of disease at DAI17, and their true leaves were slightly wrinkled and mottled greenish yellow. Watermelon plants silencing *Cla97C02G036040* (**Figure 8H**), *Cla97C02G036050* (**Figure 8I**) and *Cla97C02G036060* (**Figure 8J**) showed obvious virus symptoms at DAI13, with obvious true leaf wrinkling and large area mottled yellow.

**Figure 9:**
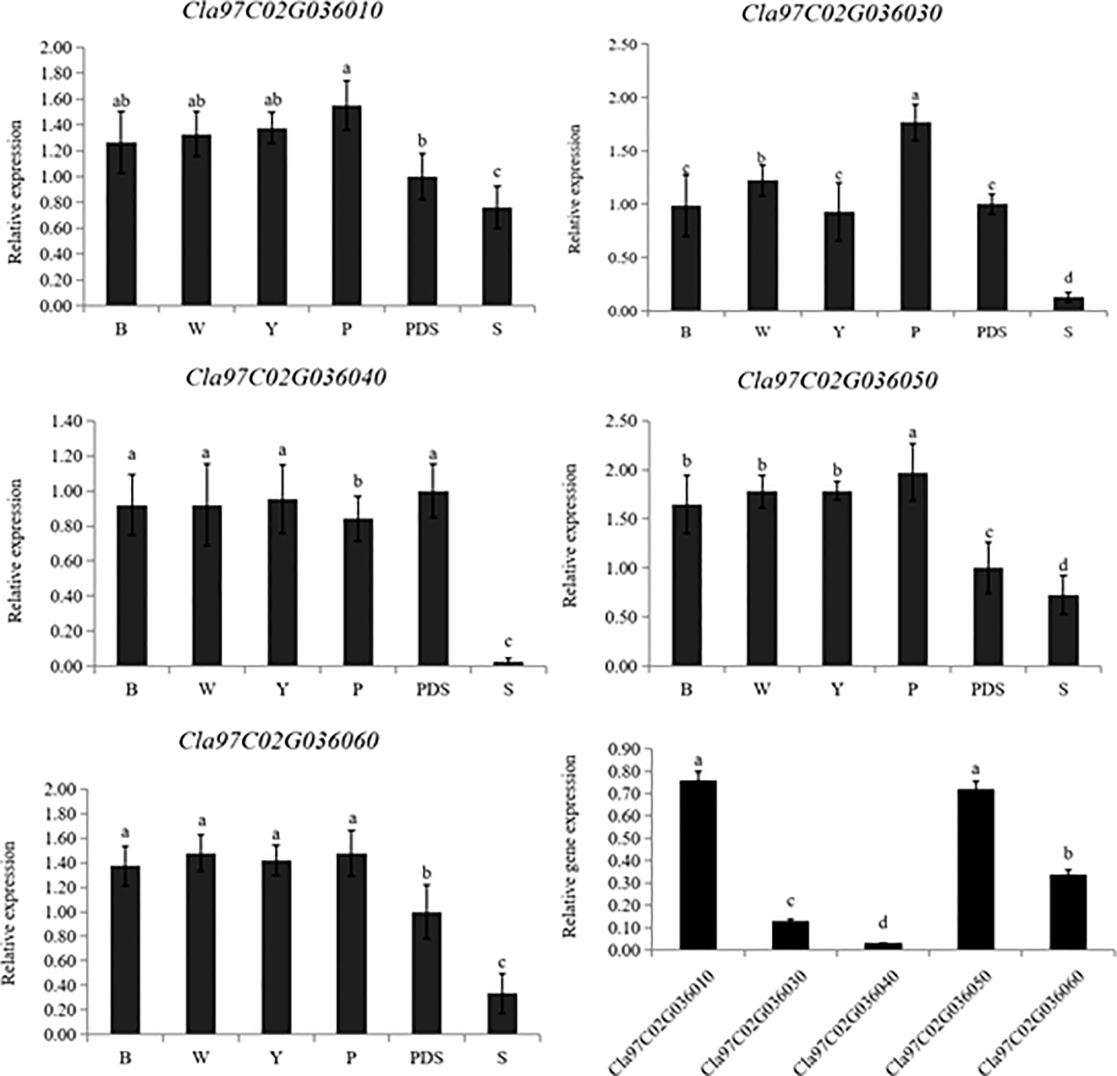
Expression levels of silenced genes. B, W, Y, P, PDS and S represents blank control, water control, YEP medium control, blank vector control, positive control and silenced genes, respectively. Small letters represent significant difference at P<0.05.

**Figure 10:**
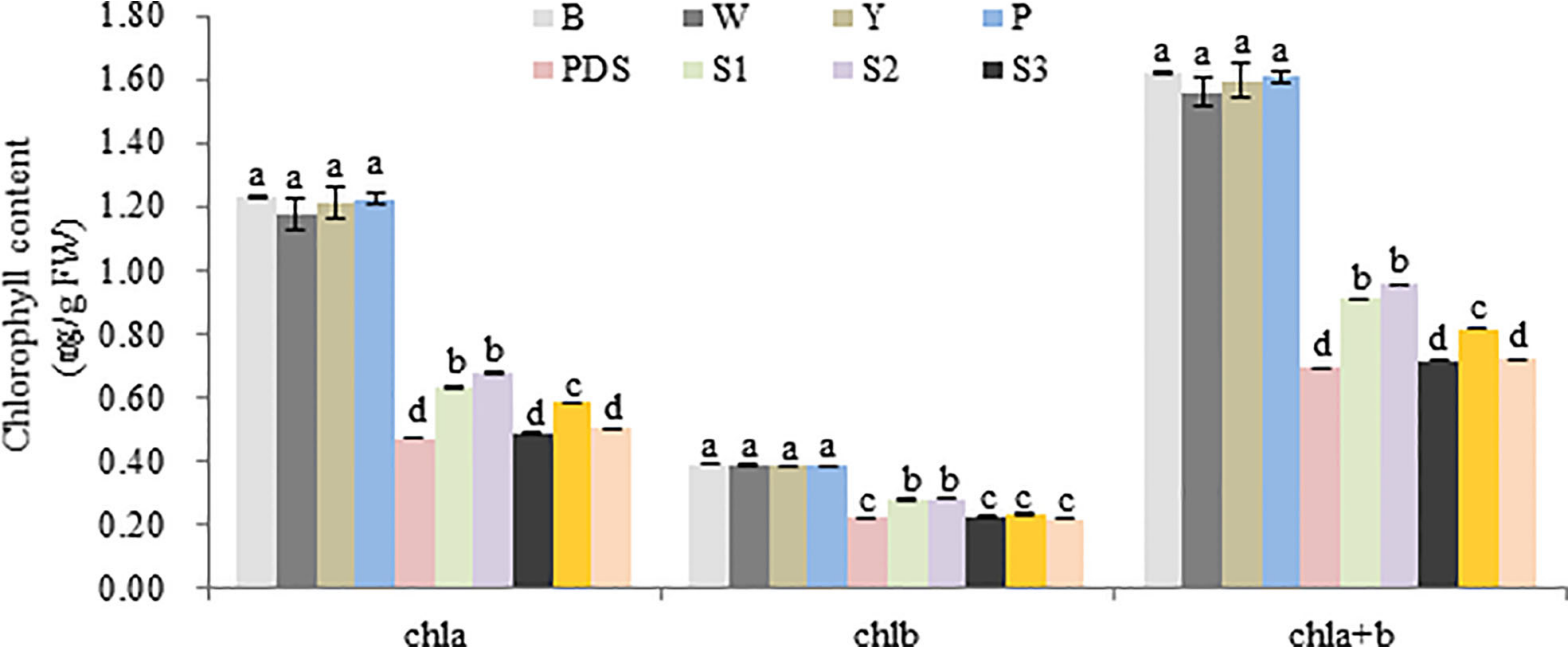
Chlorophyll content of different treatment group. B, W, Y, P, PDS, S1, S2, S3, S4,and S5 represents blank control, water control, YEP medium control, blank vector control, positive control, *Cla97C02G036010*, *Cla97C02G036030*, *Cla97C02G036040*, *Cla97C02G036050* and *Cla97C02G036060*, respectively. Small letters represent significant difference at P<0.05.

Then the expression levels of the silenced genes were detected, when compared with the control group (B, W, Y, P and PDS), their expression levels were significantly reduced. Among them, the expression of *Cla97C02G030640* decreased most sharply, which were 2.9%, 2.9%, 2.8%, 3.2% and 27% of the control group, respectively (**Figure 9**). Besides, the results of chlorophyll content of leaves with phenotype showed that there was no significant difference in chla, chlb and chla+b content among groups B, W, Y and P, while the chlorophyll contents of silenced *PDS* group was significantly lower than that of the former four groups. For the five silenced genes, the contents of chla, chlb and chla+b were significantly lower than those of B, W, Y and P control groups. Among them, the contents of chla, chlb and chla+b in silenced genes *Cla97C02G030640* and *Cla97C02G030660* were the lowest and had not significantly different from those in silenced *PDS* group (**Figure 10**).

**Figure 11:**
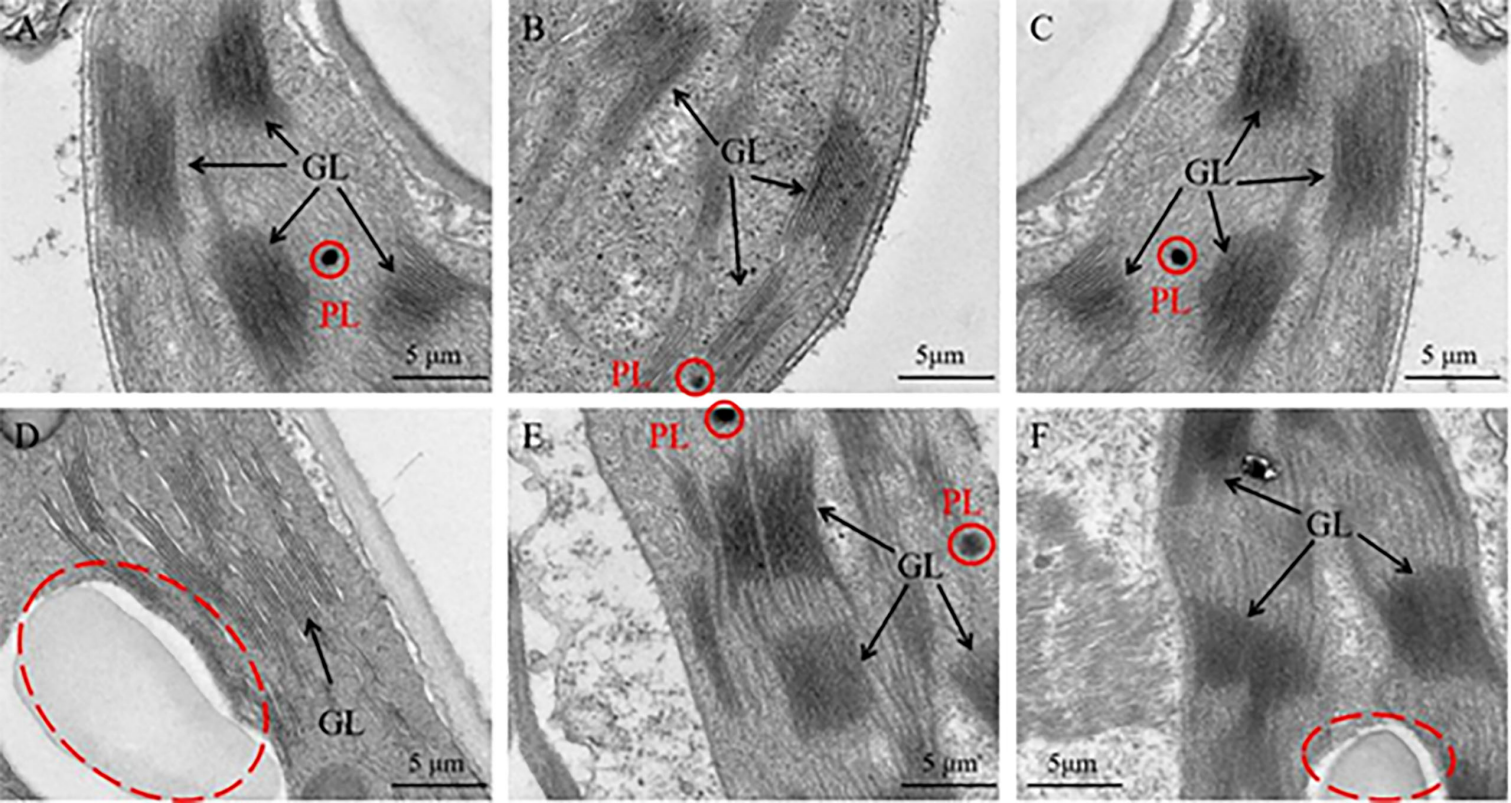
Ultrastructure of the chloroplast. **(A)** Chloroplast ultrastructure of blank control, Chloroplast ultrastructure of silencing gene **(B)** *Cla97C02G036010*, **(C)** *Cla97C02G036030*, **(D)** *Cla97C02G036040*, **(E)** *Cla97C02G036050* and **(F)** *Cla97C02G036060*, respectively. GL represents grana lamella, PL represents plastoglobuli (red circle). The scale is 5 μm.

Furthermore, the ultrastructure of chloroplast was analyzed to analyze the reasons for these phenomenon. As a result, the chloroplast ultrastructure of the silenced gene *Cla97C02G030610* (**Figure 11B**), *Cla97C02G030630* (**Figure 11C**) and *Cla97C02G030650* (**Figure 11D**) did not change compared with the blank control (**Figure 11A**), and all contained normal grana lamella (GL) and plastid globule (PL). However, the ultrastructure of silenced gene *Cla97C02G030640* and *Cla97C02G03060* was significantly changed, and the chloroplast structure may be damaged (red dotted circle area). For silenced gene *Cla97C02G030660* (**Figure 11F**), there was no PL, and PG stratification was not obvious, appearing in a fuzzy state. Especially for the silenced gene *Cla97C02G030640* (**Figure 11D**), there was no PL and no obvious PG, speculating that PG was degraded.

Taken together, these results indicated that candidate genes play an important role in causing leaf yellowing, especially *Cla97C02G030640*.

## Discussion

There are various types of leaf color mutations, and leaf yellowing was the most common phenomenon (Jin et al., 2021). Plant leaf yellowing mutants, also known as chlorophyll deficiency mutants, are usually caused by the destruction of chlorophyll synthesis or degradation pathways (Yang et al., 2014). At present, yellowing mutants have been found in rice (Zhang et al., 2017), tomato (Yao et al., 2010) and rape (Xiao et al., 2013). In this study, we reported a whole growth period leaf yellowing watermelon material *w-yl* (**Figure 1A**), which is completely different from the published watermelon leaf color mutant material (Wang et al., 2011; Haileslassie, 2020), and the leaf yellowing characteristics of *w-yl* can be stably inherited. Light can affect plant chloroplast development and chlorophyll metabolism. For example, light intensity is very important for chloroplast formation (Franck et al., 2000), which can change the proportion and content of anthocyanins or chlorophyll or carotenoids by affecting the activity of enzymes related to pigment synthesis or the expression of genes related to photosynthesis, thus causing the color change of leaves, and eventually leading to the formation of leaf color mutants (Xu et al., 2021). In cucumber, the pigment content of the post-green mutant *SC311Y* increased significantly under lower light conditions and was vulnerable to light (Zhang et al., 2022). In addition, the synthesis process of chlorophyll is regulated by many enzymes, and its activity is regulated by temperature (Yang et al., 2018). In rice, mutant *tcd9* showed abnormal chloroplasts and fewer thylakoid lamellae in albino mutant seedlings at low temperature, but the mutant showed normal green color at high temperature (Jiang et al., 2014). In *Arabidopsis*, a heat-sensitive mutant in *tsl1* is impaired in chloroplast RNA editing at high temperatures, hampering chloroplast development (Sun et al., 2020). However, under high temperature and low temperature, high light intensity and low light intensity, chlorophyll content and carotenoid content of *w-yl* had no significant difference compared with normal temperature and light intensity (**Figures 1B–F**), which suggesting that the mutant *w-yl* was non-photosensitive and non-temperature sensitive.

Previous study had confirmed that the chloroplast volume, the number of thylakoids and the number of grana lamellae in the leaves of mutant *w-yl* are smaller, which leads to a significant reduction in chlorophyll content (Ren et al., 2019). In fact, leaf yellowing mutations are usually caused by incomplete chloroplast development. For example, the yellow green leaf mutant *ygl8* in rice was caused by chloroplast dysplasia (Kong et al., 2016) The mutation of *ChlI/Chl9^pyl3^* gene in rice leaded to the formation of *pyl3* mutant with light yellow leaves. which inhibited chlorophyll synthesis, resulting in chloroplast dysplasia and leaf color variation (Hu et al., 2021). In *Brassica napus*, the chloroplast morphology of the leaf yellowing mutant *S28-y* was abnormal, with no complete grana and grana lamellae, resulting in total chlorophyll deficiency (Ge et al., 2022). A large number of studies have shown that chlorophyll is the main factor affecting leaf color phenotype, and leaf color phenotype is closely related to chlorophyll content, and the proportion of photosynthetic pigments in leaves can be directly expressed by the depth of leaf color (Chen et al., 2017; Chen et al., 2018; Su et al., 2020). Chlorophyll precursor material is the intermediate product of chlorophyll synthesis process, any step of which will influence the chlorophyll content (Wang et al., 2009) (Beale, 2005). For example, in rice leaf yellowing mutant *W1*, the process from porphobilinogen to uroporphyrinogen III was blocked, which hindered the synthesis of chlorophyll (Cui et al., 2001). In addition, Kong et al. found that the *YGL8* gene isolated and identified in *ygl8* rice yellow-green leaf mutant can encode Mg-protoIX, which plays an important role in chlorophyll synthesis by affecting the transcription level of this enzyme to change chlorophyll content (Kong et al., 2016). In Ilex × attenuata ‘Sunny Foster’, the contents of ALA, protoIX, Mg-protoIX and pchlide in green-turned leaves were significantly increased, and the chlorophyll content was also significantly higher than that in normal leaves (Huang et al., 2021). Similarly, in *Camellia sinensis* cv. Baiye1, the contents of ALA, protoIX, Mg-protoIX and pchlide were higher in green leaves, and the chlorophyll content was also significantly higher than that in albino leaves (Wang et al., 2008). Similar results were obtained in this study, such as the contents of ALA, protoIX, Mg-ProtoIX and pchlide in *w-yl* were significantly lower than those in normal leaves ZK, indicating that the low chlorophyll content in *w-yl* may be due to the low content of chlorophyll precursors.

There are many kinds of leaf color mutations, and the genetic rules of different mutations vary greatly, which may be nuclear inheritance or cytoplasmic inheritance. For example, rice (Sun et al., 2017), maize (Wang et al., 2018) wheat (Jiang, 2018), cucumber (Gao et al., 2016), rape (Wang, 2014), tomato (Guo, 2017) and cabbage (Zhang, 2017) are controlled by single or two pairs of recessive nuclear genes. In watermelon, Zhang et al. (1996) proved that albino leaf color mutation was controlled by a pair of recessive alleles (*jaja*). Provvidenti (1994) found that the watermelon leaf color mottle mutation was controlled by a pair of recessive genes (*slv*), and the F_2_ population showed a normal:mottled separation ratio of 3:1. Rhodes (1986) found that the post-green mutation was controlled by a recessive gene (*dgdg*). The data in this study indicated that *w-yl* is controlled by a pair of recessive nuclear genes. However, the results of mapping indicated that *w-yl* may have DNA fragment deletion compared to ZK, resulting in *Cla97C02G036010*, *Cla97C02G036030*, *Cla97C02G036040*, *Cla97C02G036050* and part of *Cla97C02G036060* in the interval between InD14,179,011 and InD16,396,362 loss the gene function. Chloroplast genome gene loss is relatively common in nature (Dong, 2012). Studies have shown that the most frequent microstructural changes in the chloroplast genome are insertions and deletions, and have a bias for deletions (Gao et al., 2010). In angiosperms, *rpl22, rpl23*, *rpl32*, *rpl33*, *rps16*, *accD*, *psaI*, *ycf4*, *ycf1*, *ycf2 and infA* were lost in some taxa. Among them, *ycf1*, *ycf2* and *accD* genes were lost in the whole *Gramineae* (Guisinger et al., 2010) and some species in *Solanaceae (* Bruni et al., 2010 *)*.

In this study, the gene *Cla97C02G036060* encoded the protein Ycf2. NAD-malate dehydrogenase contained in the Ycf2/FtsHi complex is a key enzyme for ATP production in chloroplasts or non-photosynthetic plastids in the dark (Kikuchi et al., 2018), and is necessary for photosynthetic growth (Parker et al., 2016). The *Ycf2* gene is the largest plastid gene in angiosperms (Huang et al., 2010). It plays an important unknown function in higher plants and is indispensable (Drescher et al., 2000), which can response to biotic and abiotic stresses in plants (Durante et al., 2009) and improve plant cold tolerance (Bernardi et al., 2015). Gene *Cla97C02G036050* encoded a DnaJ-like B subfamily protein, which is a type of heat shock protein (Sun et al., 2018). Its homologous proteins can increase the activity of phytoene synthase in plastids (Zhou et al., 2015), participate in the process of white body differentiation into chloroplasts under light (Shimada et al., 2007), and protect plant photosystem II under heat stress (Wang and Luthe, 2003). For the gene *Cla97C02G036040*, it encoded a protein that containing the DUF679 domain. DUF (domain of unknown function) refers to a protein family with unknown functional domains, which is involved in regulating plant growth and development, plant defense mechanism and plant stress response in plants (Finn et al., 2016; Wang et al., 2022). In *Arabidopsis*, all members of DUF579 family can affect the development of xylan in plant cell wall hemicellulose (Temple et al., 2019), DUF761 is involved in regulating the growth and development of plant vegetative organs (Zhang et al., 2019), DUF642 protein is a specific protein of seed plants, which is associated with cell wall synthesis (Vázquez-Lobo et al., 2012). In cucurbit crops, there are few studies on DUF domain, mainly focusing on the disease resistance of cucumber (Liu et al., 2010; Qin et al., 2018; Wang et al., 2018).

## Data availability statement

The datasets presented in this study can be found inonline repositories. The names of the repository/repositories and accession number(s) can be found below: https://www.ncbi.nlm.nih.gov/, PRJNA872830.

## Author contributions

JL, DS, YZ, and GY designed the experiments. YZ, GY, YW, GA, and WL provided experimental methods. YZ and GY performed the research and analyzed the data and wrote the manuscript. JL and DS reviewed the manuscript. All authors contributed to the article and approved the submitted version.

## Funding

This research was supported by the National Natural Science Foundation of China (32102395), Natural Science Foundation of Xinjiang Uygur Autonomous Region (2020D01A136), Agricultural Science and Technology Innovation Program (CAAS-ASTIP-2022-ZFRI), the China Agriculture Research System of MOF and MARA (CARS-25), and Joint Key Project of Agricultural Fine Variety in Henan Province (20220100001).

## Conflict of interest

The authors declare that the research was conducted in the absence of any commercial or financial relationships that could be construed as a potential conflict of interest.

## Publisher’s note

All claims expressed in this article are solely those of the authors and do not necessarily represent those of their affiliated organizations, or those of the publisher, the editors and the reviewers. Any product that may be evaluated in this article, or claim that may be made by its manufacturer, is not guaranteed or endorsed by the publisher.
