# Supplementary material for: Mapping and functional verification of leaf yellowing genes in watermelon during whole growth period
Source: Front Plant Sci. 2022 Oct 19;13:1049114. doi: 10.3389/fpls.2022.1049114 (PMC9627507; doi:10.3389/fpls.2022.1049114)
Supplement: Supplementary file 5 [file DataSheet_1.doc]

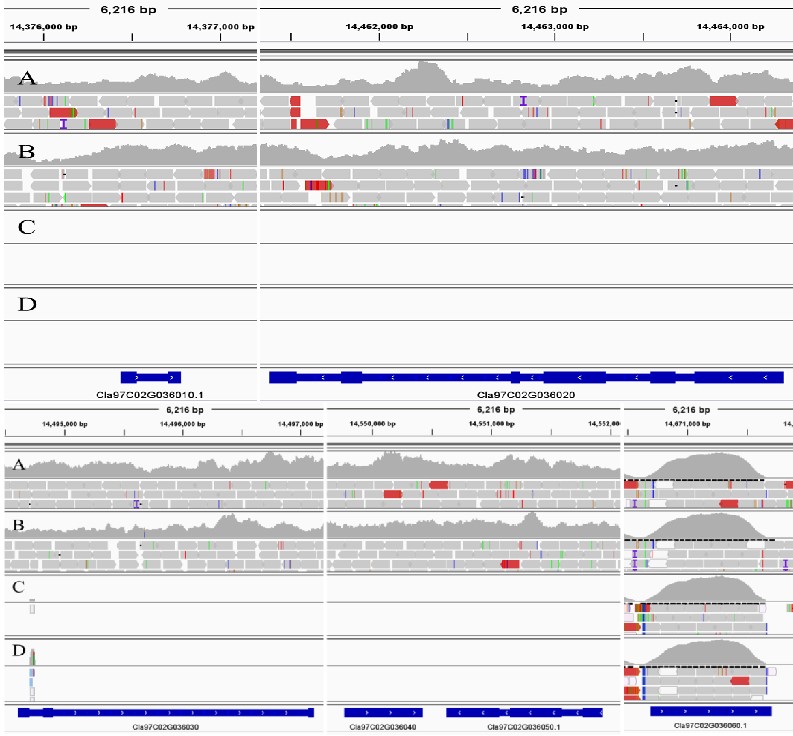


Figure S1. The position of genes in the chromosome 2. A represents ZK F_2_ mixed pool; B represents ZK parent pool; C represents *w-yl* parent pool; D represents *w-yl* F_2_ mixed pool.
